# An in vitro collagen perfusion wound biofilm model; with applications for antimicrobial studies and microbial metabolomics

**DOI:** 10.1186/s12866-019-1682-5

**Published:** 2019-12-30

**Authors:** Elisabeth A. Slade, Robin M. S. Thorn, Amber Young, Darren M. Reynolds

**Affiliations:** 1 Centre for Research in Biosciences, University of the West of England, Bristol, UK; 2 Scar Free Foundation Centre for Children’s Burns Research, Bristol Royal Hospital for Children, Bristol, UK; 3 University of the West of England, Frenchay Campus, Coldharbour Lane, Bristol, BS16 1QY England

**Keywords:** Biofilm, Collagen, Wound, In vitro model, Volatile metabolite, *Pseudomonas aeruginosa*

## Abstract

**Background:**

The majority of in vitro studies of medically relevant biofilms involve the development of biofilm on an inanimate solid surface. However, infection in vivo consists of biofilm growth on, or suspended within, the semi-solid matrix of the tissue, whereby current models do not effectively simulate the nature of the in vivo environment. This paper describes development of an in vitro method for culturing wound associated microorganisms in a system that combines a semi-solid collagen gel matrix with continuous flow of simulated wound fluid. This enables culture of wound associated reproducible steady state biofilms under conditions that more closely simulate the dynamic wound environment. To demonstrate the use of this model the antimicrobial kinetics of ceftazidime, against both mature and developing *Pseudomonas aeruginosa* biofilms, was assessed. In addition, we have shown the potential application of this model system for investigating microbial metabolomics by employing selected ion flow tube mass spectrometry (SIFT-MS) to monitor ammonia and hydrogen cyanide production by *Pseudomonas aeruginosa* biofilms in real-time.

**Results:**

The collagen wound biofilm model facilitates growth of steady-state reproducible *Pseudomonas aeruginosa* biofilms under wound like conditions. A maximum biofilm density of 10^10^ cfu slide^− 1^ was achieved by 30 h of continuous culture and maintained throughout the remainder of the experiment. Treatment with ceftazidime at a clinically relevant dose resulted in a 1.2–1.6 log reduction in biofilm density at 72 h compared to untreated controls. Treatment resulted in loss of complex biofilm architecture and morphological changes to bacterial cells, visualised using confocal microscopy. When monitoring the biofilms using SIFT-MS, ammonia and hydrogen cyanide levels peaked at 12 h at 2273 ppb (±826.4) and 138 ppb (±49.1) respectively and were detectable throughout experimentation.

**Conclusions:**

The collagen wound biofilm model has been developed to facilitate growth of reproducible biofilms under wound-like conditions. We have successfully used this method to: (1) evaluate antimicrobial efficacy and kinetics, clearly demonstrating the development of antimicrobial tolerance in biofilm cultures; (2) characterise volatile metabolite production by *P. aeruginosa* biofilms, demonstrating the potential use of this method in metabolomics studies.

## Background

It is widely accepted that bacteria commonly exist in sessile communities known as biofilms, rather than as individual free swimming cells [1]. Biofilms are implicated in a range of clinically relevant infections including lung infection in cystic fibrosis, endocarditis, osteomyelitis, acute burn infection and chronic wound infection [2, 3]. Established biofilms are typically highly tolerant to antimicrobials and the host immune response [4]. The extracellular polymeric substance (EPS) protects bacterial cells within the biofilm by providing a physical barrier which decreases penetration of antimicrobials and agents of the host immune system [5, 6]. Furthermore, this biofilm phenotype is often coupled with a reduced metabolic activity and growth rate, typically seen within the interior of biofilm communities, which reduces the susceptibility to those antimicrobials which target such processes [7, 8]. A recent review of clinical studies conducted between 2008 and 2015, concluded that biofilms were present in 78.2% of chronic wounds [9], indicating the important role that biofilms play in the development of a chronic wound state. Detailed investigation of the distribution of infecting organisms within chronically infected wounds has demonstrated differences in the depth of microbial biofilm aggregates embedded within the wound bed [10–13]. For example, one study demonstrated the presence of *Pseudomonas aeruginosa* micro colonies embedded deeper within the wound bed than those of *Staphylococcus aureus* which were found closer to the wound surface [12]. The role of biofilms in the infection of acute wounds has been less well characterized, however biofilm formation has been observed in animal models of acute burn wound and surgical site infection [11, 14, 15].

In vitro studies of biofilms employ one of two approaches; a closed multi-well plate based model, or an open flow system with the continuous perfusion of nutrient into the model and waste products continuously exiting the system [16]. Both of these approaches often involve the development of biofilms on solid surfaces, usually plastic or glass. Microbial infection in vivo consists of biofilm growth on the surface of, or suspended within, the semi-solid matrix of the tissue, unless adhered to an implanted medical device or catheter [17]. Collagen based gel matrices have been used as a substratum for culturing biofilms in vitro, in an attempt to closely simulate the semi-solid nature of the wound [18–22]. However, a limitation of these closed systems, is that they do not simulate the replacement of nutrients and moisture that occur within the wound bed due to the production of exudate, which provides the continuous flux of nutrients available to the biofilm during formation and growth [23, 24].

To further our understanding and knowledge of wound infection processes, a wound biofilm model is required that better simulates the wound environment. This could be achieved by the combination of a continuous flow biofilm model system with a semi-solid ‘wound-like’ growth substrate and a more representative growth media. We describe an in vitro method for culturing wound associated microorganisms within a collagen wound biofilm model, combining a drip flow reactor system (Biosurface technologies, MZ, USA) with a three-dimensional type I collagen gel growth matrix and continuous perfusion of a previously developed simulated wound fluid (SWF) [18, 21, 25, 26]. To demonstrate the application of this method, it has been utilized to study the antimicrobial kinetics of ceftazidime, when used to treat *P. aeruginosa* biofilms during early or late stage development. *P. aeruginosa* is among the most commonly isolated pathogens from both chronic and acute burn wound infections [27, 28] and biofilms have been shown to rapidly result in systemic infection in a mouse model of acute burn infection [14]. Ceftazidime is considered a first choice antipseudomonal antibiotic [29, 30] for treatment when there is a high risk of systemic infection developing from an infected wound. To further demonstrate the potential of this novel model, a preliminary investigation of microbial metabolomics in relation to microbial biofilm development was undertaken using selected ion flow tube mass spectrometry (SIFT-MS). Ammonia and hydrogen cyanide have previously been reported as important potential diagnostic metabolites detected in the headspace of *P. aeruginosa* liquid cultures in vitro [31]*,* and in *P. aeruginosa* infections in vivo [32, 33], through analysis of the exhaled breath of cystic fibrosis patients. Hydrogen cyanide is generated through decarboxylation of the amino acid glycine by the membrane bound hydrogen cyanide synthase enzyme [34], ammonia is produced by the metabolism of nitrogen containing compounds including hydrogen cyanide [31]. Real-time monitoring of bacterial volatile metabolites, has gained momentum in recent years as a potential rapid diagnostic tool [32, 35–41]. The novel collagen wound biofilm model reported here, allows the development of this diagnostic approach in the context of wound infection. For example by the detection of volatile metabolite profiles emitted by biofilm cultures, produced under conditions which closely simulate the wound environment.

## Results

### Characterization of the in vitro collagen perfusion wound biofilm model

**Fig. 1:**
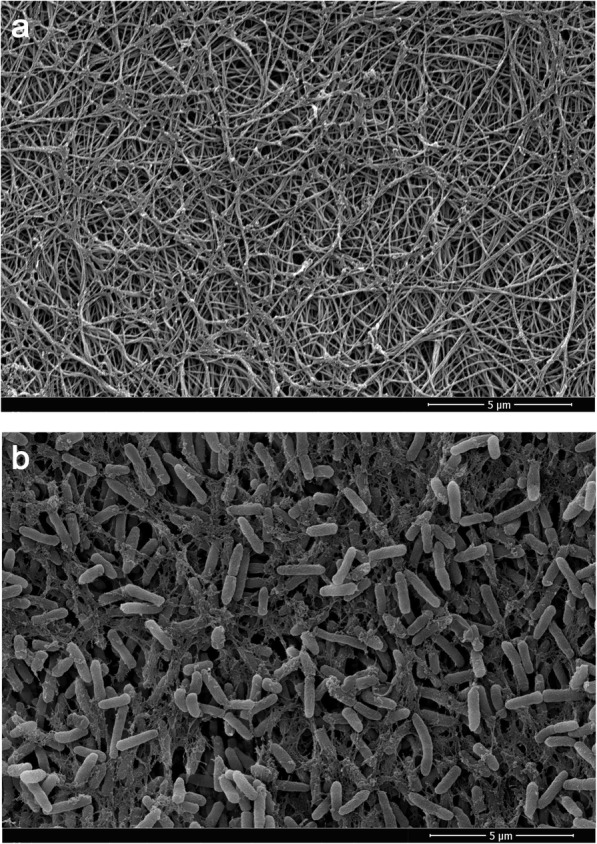
Scanning electron micrographs of **a** collagen gel and **b** *Pseudomonas aeruginosa* NCIMB 10548 biofilm cultured on a collagen gel matrix

The un-inoculated collagen growth matrix, which polymerizes at 37 °C to form a hydrated three-dimensional semi-solid gel layer on the surface of the microscope slide coupons, was imaged using scanning electron microscopy (SEM). The fixation and dehydration process required for preparation of samples for SEM results in collapse of the three-dimensional structure, although the mesh-like network of long collagen fibers remains clearly visible (Fig. 1a). SEM imaging of a *P. aeruginosa* biofilm cultured on the collagen gel growth matrix shows a dense layer of microbial cells masking the collagen fibers below. Multiple layers of *P. aeruginosa* are visible, as is the dehydrated biofilm extracellular polymeric substances (EPS) which can be see connecting adjacent bacterial cells (Fig. 1b).

The developed novel collagen wound biofilm model system supports growth of reproducible *P. aeruginosa* biofilms during both early and late stage development. A maximum biofilm density of 10^10^ cfu slide^− 1^ (dictated by the experimental conditions) was achieved by 30 h of continuous culture.

**Fig. 2:**
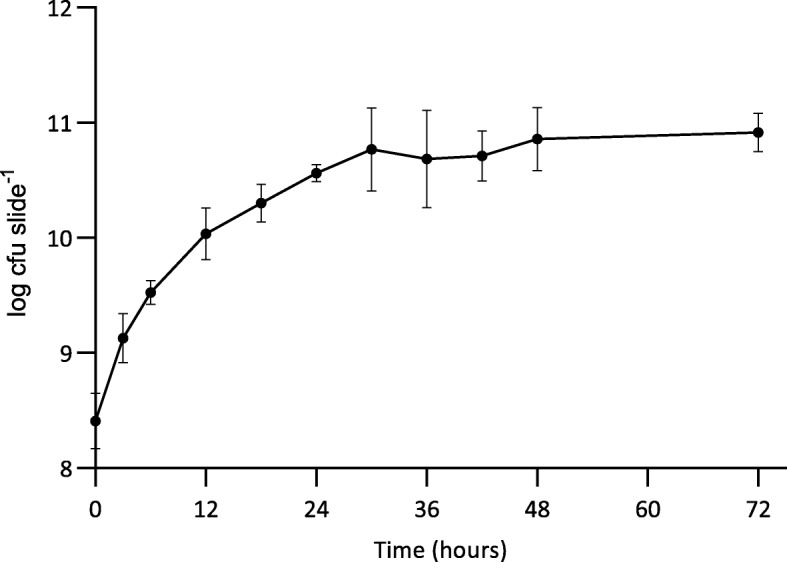
*Pseudomonas aeruginosa* NCIMB 10548 biofilm density when grown in the collagen wound biofilm model system over 72 h at 33 °C (*n* = 3 per time point; mean ± SD). An approximately steady-state at 6.0 × 10^10^–8.0 × 10^10^ cfu slide^− 1^ was maintained from 30 h to the end of experimentation at 72 h

**Fig. 3:**
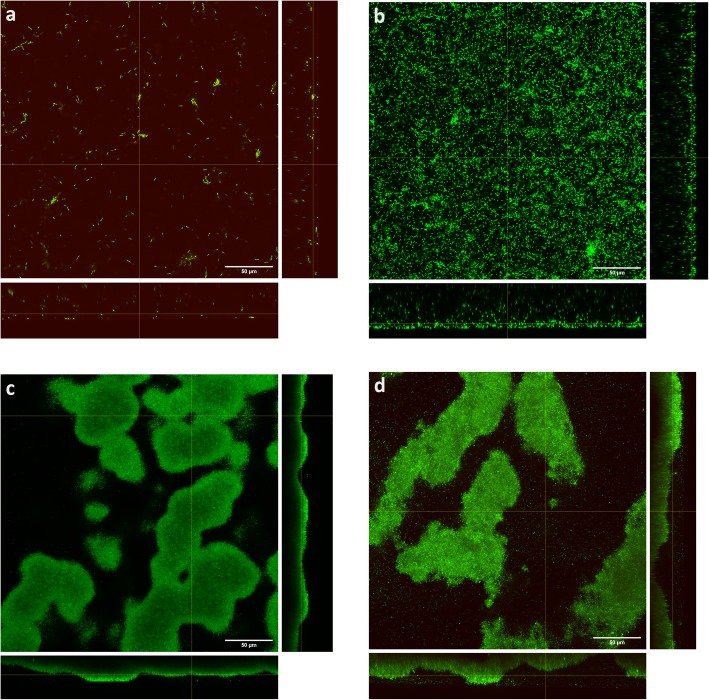
Confocal scanning laser micrographs of *Pseudomonas aeruginosa* NCIMB 10548 biofilms grown in the collagen wound biofilm model. Sampled after **a** 6 h, **b** 12 h, **c** 24 h and **d** 48 h of continuous culture. Main panels show XY plane, right panels show YZ slice and bottom panels show XZ slice. Green Syto 9 staining indicates live bacterial cells and red propidium iodide staining indicates dead bacterial cells

Figure 2 shows growth of *P. aeruginosa* NCIMB 10548 biofilms over 72 h at 33 °C in the collagen wound biofilm model. The maximum density was maintained at an approximately steady-state of 6.0 × 10^10^–8.0 × 10^10^ cfu slide^− 1^, from 30 h until the end of experimentation (72 h) and equates to a microbial burden per gram of collagen of 4 × 10^10^–5.3 × 10^10^ cfu g^− 1^. In addition to sampling for enumeration, biofilms were sampled after 6, 12, 24 and 48 h of continuous culture for confocal microscopy, to determine the structural arrangement of bacterial cells and biofilm architecture during growth and development (Fig. 3). The biofilm sampled at 6 h consisted of sparsely arranged *P. aeruginosa* cells with some small aggregates (Fig. 3a), whereas at 12 h the cell density had increased and some larger aggregates were visible (Fig. 3b). At 24 h the density of the bacterial cells had again increased, in line with the enumeration data (Fig. 2), whereby more complex structural formations and the development of surface protrusions and channels can be seen (Fig. 3c). At 48 h, the biofilm architecture within the maturing biofilm shows evidence of increased variability in surface topography, as described within other biofilm studies [42–44].

### Application of the collagen wound biofilm model for studying antimicrobial kinetics

**Fig. 4:**
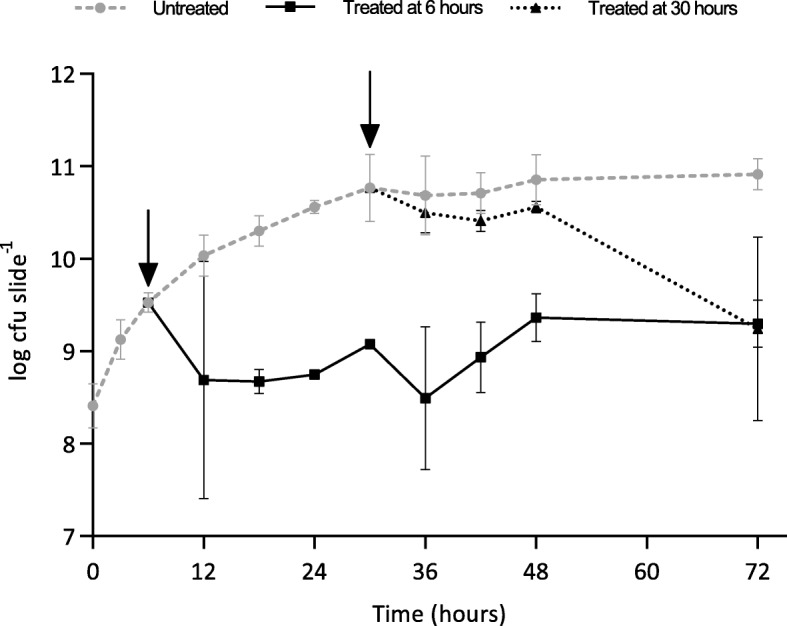
Treatment of *Pseudomonas aeruginosa* NCIMB 10548 biofilms with ceftazidime at 40 mg L^-1^, showing antimicrobial kinetics when used against established maturing biofilms (30 h) and early stage biofilms (6 h), compared to untreated controls. (*n* = 3 per time point per condition; mean ± SD). Arrows indicate treatment start times. Treatment started at 30 h resulted in a 1.2 log reduction in total biofilm density at 72 h, compared to untreated controls (*p* < 0.05). Treatment started at 6 h resulted in a 1.6 log reduction in total biofilm density at 72 h, compared to untreated controls (*p* < 0.01). No significant difference (*p* > 0.05) between treated groups at 72 h

To demonstrate the application of this novel method the collagen wound biofilm model was used to investigate the antimicrobial activity of antibiotic therapy against biofilms at a clinically relevant dose. To confirm susceptibility of *P. aeruginosa* NCIMB 10548 to ceftazidime, planktonic minimum inhibitory concentration (MIC) values were determined (*n* = 3) using a standard microdilution multi-well plate assay [45]; the MIC was determined to be 2.0 mg L^− 1^. The clinical breakpoint for ceftazidime against *P. aeruginosa* according to the European Committee on Antimicrobial Susceptibility testing is 8 mg L^− 1^ [46]. Hence, the MIC of 2.0 mg L^− 1^, indicates that this strain is considered sensitive to ceftazidime. Once susceptibility of the strain was confirmed the efficacy of ceftazidime against *P. aeruginosa* biofilms was investigated using the collagen wound biofilm model. A target serum concentration of 40 mg L^− 1^ for continuous infusion of ceftazidime is recommended for effective treatment of *P. aeruginosa* infections in vivo [29, 30, 47], hence this drug concentration was selected to challenge the *P. aeruginosa* biofilms in the collagen wound biofilm model. Figure 4 shows the change in biofilm density resulting from ceftazidime treatment at 40 mg L^− 1^, compared to normal growth conditions. Treatment was started after either 6 h or 30 h of continuous culture, to enable comparison of treatment efficacy on the early stage of biofilm formation and on established steady state maturing *P. aeruginosa* biofilms. When treatment was initiated at the early stage of biofilm formation, the total biofilm density decreased for the first 12 h of treatment to approximately 5 × 10^8^ cfu slide^− 1^ (1.63 log reduction compared to untreated controls; *p* < 0.01). The biofilm density remained stable at the subsequent sampling time, but increased over the following 24 h to reach a density of 2 × 10^9^ cfu slide^− 1^. This density was maintained until the final sampling time (72 h of continuous culture) resulting in a significant 1.6 log reduction compared to untreated controls (*p* < 0.05) at the end of experimentation. During treatment of established maturing biofilms, the biofilm density gradually decreased ultimately resulting in a mean biofilm density of 5.5 × 10^9^ cfu slide^− 1^ at 72 h (a significant 1.2 log reduction compared to the untreated control; *p* < 0.05). When comparing treatment initiated at the early stage of biofilm development or on established maturing biofilms, although both treatment regimens exhibited a significant antimicrobial effect, there was no significant difference in biofilm density between these treatment groups after 72 h of continuous culture.

**Fig. 5:**
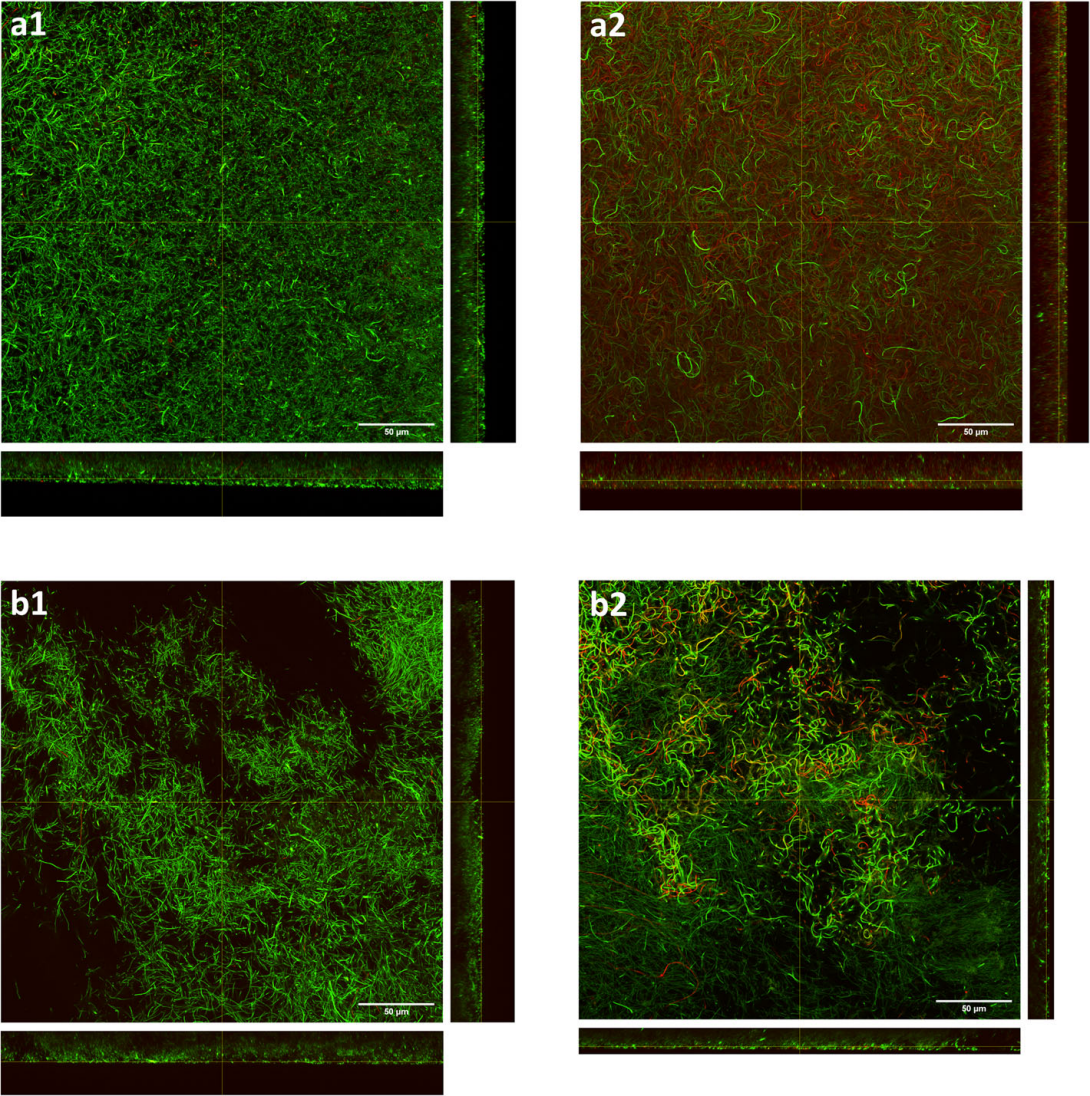
Confocal scanning laser micrographs of *Pseudomonas aeruginosa* NCIMB 10548 biofilms treated with ceftazidime at 40 mg L^− 1^. Top panels, treatment initiated at 6 h (early stage of biofilm formation); (**a1**) sample imaged following 18 h exposure to treatment and (**a2**) 42 h exposure to treatment. Bottom panels, treatment initiated at 30 h (maturing biofilm); (**b1**) biofilm sampled following 18 h exposure to treatment (48 h) and (**b2**) 42 h exposure to treatment. Main panels show XY plane, right panels show YZ slice and bottom panels show XZ slice. Green Syto 9 staining indicates live bacterial cells and red propidium iodide staining indicates dead bacterial cells

Figure 5 shows confocal scanning laser micrographs of *P. aeruginosa* biofilms treated with 40 mg L^− 1^ ceftazidime. Samples for both treatment regimens were imaged after both 18 h and 42 h of exposure to the ceftazidime; this corresponds to a total culture time of 24 h and 48 h for the samples where antibiotic treatment was started at the early stage of biofilm formation, and 48 h and 72 h total culture time for the samples where treatment was started on established biofilms. Samples imaged following 18 h of exposure to ceftazidime at 40 mg L^− 1^ (Fig. 5; a1 & b1) both show some elongation of *P. aeruginosa* cells. Within the confocal z-stack, it was observed that this was most pronounced in the bacterial cells nearest to the surface of the biofilms, whereas cells deeper within the biofilm were morphologically more similar to the untreated sample (Fig. 3). After 42 h of antibiotic exposure, it was observed that there was a high proportion of elongated bacterial cells (Fig. 5; a2 & b2). The biofilm appears less densely packed with clear spaces between the tangles of filamentous cells, compared to untreated biofilms shown in Fig. 3. By comparing untreated (Fig. 3c) and treatment of early stage biofilms (Fig. 5; a1 and a2) it is clear that the ceftazidime treatment prevents the development of complex biofilm architecture. In addition, when comparing untreated (Fig. 3d) and the treatment of established biofilms (Fig. 5; b1), it is evident that ceftazidime treatment has resulted in the collapse of the characteristic three-dimensional biofilm structure. Demonstrating the effect of ceftazidime on the complex biofilm structure, as well as the morphology of individual bacterial cells.

### Application of the collagen wound biofilm model for studying biofilm metabolomics

**Fig. 6:**
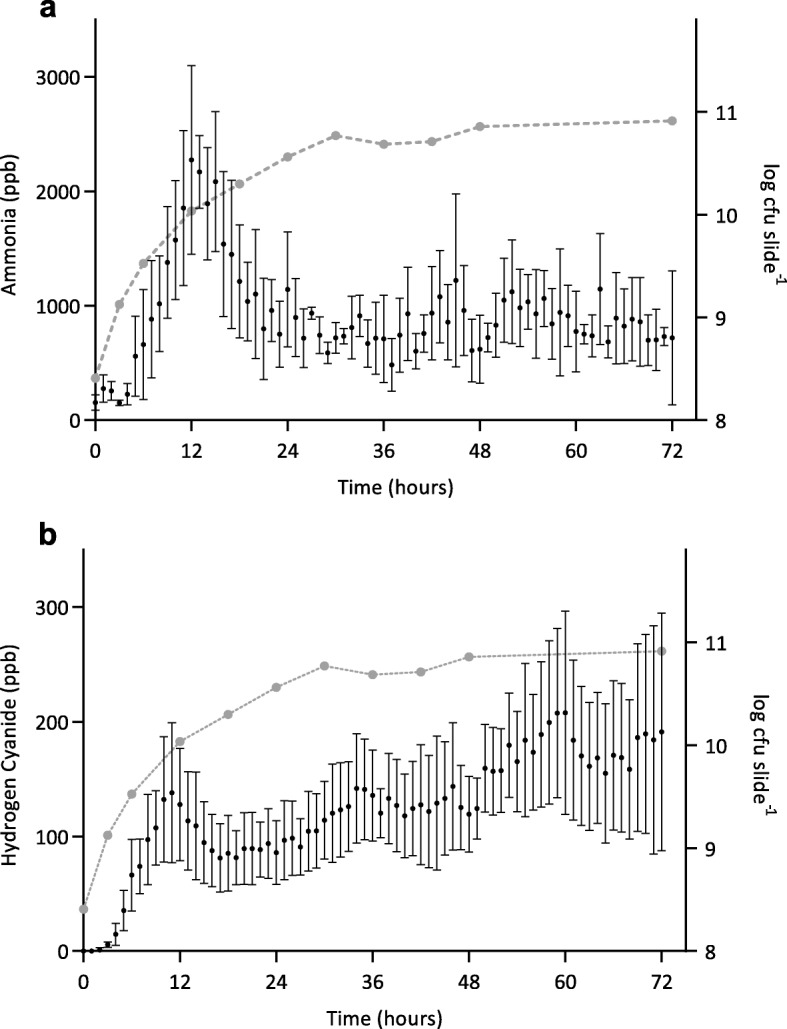
Detection of volatile metabolites ammonia and hydrogen cyanide during growth and development of *Pseudomonas aeruginosa* NCIMB 10548 biofilms in the collagen wound biofilm model. **a** Ammonia concentration (ppb) detected in the headspace of *P. aeruginosa* biofilms for 72 h (black); left Y axis (*n* = 4; mean ± SD). Mean biofilm density over 72 h (grey): right Y axis. **b** Hydrogen cyanide concentration (ppb) detected in the headspace of *P. aeruginosa* biofilms for 72 h (black); left Y axis ( = 4; mean ± SD). Mean biofilm density over 72 h (grey): right Y axis

Selected ion flow tube mass spectrometry was used to monitor headspace concentrations of volatile metabolites in real-time. Detection of ammonia and hydrogen cyanide was chosen to demonstrate the capability of analyzing volatile metabolites produced by developing *P. aeruginosa* biofilms in the collagen wound biofilm model. Biofilm headspace was sampled and analyzed repeatedly using the SIFT-MS instrument throughout the 72 h duration of biofilm growth and development. Figure 6 shows the concentration of ammonia and hydrogen cyanide respectively, detected in the headspace of *P. aeruginosa* biofilm cultures. The concentration of both compounds (ppb) had an initial peak at 12 h, with a mean concentration of 2273 ppb for ammonia and 138 ppb for hydrogen cyanide. The hydrogen cyanide concentration then dropped to a mean concentration of 81 ppb at 18 h before again increasing gradually to a peak of 191 ppb. The concentration of ammonia also dropped, with a mean concentration of 797 ppb recorded at 21 h and then continued to fluctuate between approximately 600 and 1200 ppb for the remaining duration of analysis. The concentration of both ammonia and hydrogen cyanide remained at levels detectable using SIFT-MS throughout the duration of growth and development of *P. aeruginosa* biofilms within the collagen wound biofilm model.

## Discussion

The dermis of human skin is primarily composed of the protein collagen [48]. Type I collagen is the most abundant type found in human skin and is distributed throughout all layers of the dermis [49]. We have successfully developed a dynamic collagen wound biofilm model utilising a three-dimensional collagen gel growth matrix comprised of a mesh of polymerized type I collagen fibres (Fig. 1), to simulate the semi-solid wound environment found in vivo*.* Continuous perfusion with SWF provides replacement of moisture and nutrients, and removal of waste products, that would be provided by the flow of exudate within infected wounds [50]. The simulated wound fluid provides a similar range of nutrients as exudate, a high protein fluid that leaks from the blood vessels in response to inflammation associated with wound healing processes [51]. Biochemical analysis of wound fluid has shown that a similar range of constituents are present when compared to serum, with concentrations in would fluid at lower levels than in serum for the majority of components [52]. This suggests use of a simulated wound fluid comprised of 50% serum will provide a suitable range of nutrients, many of which will be at biologically relevant concentrations. We have validated the growth of *P. aeruginosa* with this method and demonstrated that it enables culture of reproducible steady state biofilms at a density greater than that considered to be the critical threshold of clinically relevant wound infection. A density of 10^5^ cfu g^− 1^ tissue is considered the critical threshold indicative of clinically relevant wound infection [53–55], with a bioburden of between 10^9^ and 10^11^ cfu g^− 1^ tissue identified from infected wounds with the heaviest bacterial loads [54, 56]. Hence, the density achieved within the collagen wound biofilm model represents an appropriate bacterial load, reflective of a challenging clinical situation for assessing antimicrobial efficacy. Characterisation of the collagen wound biofilm model has focused on cultivation of *P. aeruginosa* NCIMB 10548, a commercially available strain originally isolated from an infected wound. However, future studies could apply the collagen wound biofilm model to investigate the growth rate and susceptibility of clinical isolates of *P. aeruginosa* and other wound associated species.

MIC assays are routinely used clinically to determine the susceptibility of bacterial isolates to the antibiotic treatments available. However, as demonstrated within this study, this is unlikely to predict the effectiveness of antimicrobials against biofilms and hence their therapeutic effectiveness against biofilm infections. To demonstrate the application of this model system for evaluating the efficacy and kill kinetics of antimicrobial agents, both developing and established *P. aeruginosa* biofilms were challenged with the cephalosporin antibiotic ceftazidime. There was no significant difference (*p* > 0.05) in the final biofilm density after 72 h of continuous culture, when comparing the two treatment start times. Both treatment strategies resulted in a significant reduction of total biofilm density at 72 h compared to the untreated biofilm. Although a statistically significant reduction in viable *P. aeruginosa* biofilm density was demonstrated, the remaining mean biofilm density of 2.2 × 10^9^ and 5.5 × 10^9^ cfu slide^− 1^ for the two treatment regimens equates to 1.5 × 10^9^ and 3.6 × 10^9^ cfu g^− 1^ of collagen ‘tissue’ respectively. This density remains vastly in excess of the 10^5^ cfu g^− 1^ of ‘tissue’, used as the critical threshold indicative of invasive infection and risk of sepsis in studies of quantitative microbiological analysis of wound biopsy samples [54, 55]. It has previously been reported that there are differences between the planktonic MIC and the concentration of ceftazidime (and a range of other antimicrobials) required to eradicate biofilms, with concentrations 1000-fold greater than the MIC unable to eradicate *P. aeruginosa* biofilms within a static model [57]. Using the collagen wound biofilm model, we have shown the time course of the development of such tolerance for the first time under wound like conditions in vitro, and visualized the effect of drug treatment on the biofilm structure. Ceftazidime treatment resulted in distinct morphological changes in the general biofilm architecture as well as the discrete bacterial cells. This elongation response by *P. aeruginosa* to β-lactam antibiotics, including ceftazidime, has been described previously and was observed to result in cell lysis and a reduction in viable cells in planktonic culture [58]. It has been determined that inhibition of penicillin binding protein 3 (PBP3) by β-lactam antibiotics is responsible for causing filamentation in *P. aeruginosa*. This has been demonstrated by deletion of the gene required for PBP3 expression and comparison of the resulting morphological changes to those seen when *P. aeruginosa* was exposed to sub-lethal concentrations of β-lactam antibiotics [59]. This phenomena has also been observed in response to β-lactam exposure in *Escherichia coli* [60]*,* where cell elongation is reported to be the first of a four step process leading to eventual cell lysis.

The developed method facilitates investigation of microbial metabolomics, including volatile metabolites produced by bacterial species associated with causing clinically relevant wound infection [38]. Selected ion flow tube mass spectrometry was used for real-time detection of the volatile compounds ammonia and hydrogen cyanide in the collagen wound biofilm model. Monitoring of volatile compounds during biofilm growth and development from a perfusion biofilm model has not been reported previously. In the collagen wound biofilm model, the concentration of both compounds increased for approximately the first 12 h, corresponding to the most rapid increase in biofilm density. Subsequently, the concentration of both compounds decreased from 12 to 24 h. Investigations of real-time production of hydrogen cyanide and ammonia from reference strains and clinical isolates of *P. aeruginosa* in liquid culture indicated a peak in hydrogen cyanide concentration at the transition to stationary phase only [31]. Interestingly, the drop in concentration of both volatile compounds between 12 and 24 h, corresponds with distinct changes in the arrangement of bacterial cells and development of the biofilm structures observed by confocal microscopy (Fig. 3), as well as a decrease in the rate at which the biofilm density was increasing. When the biofilm density had stabilized within the collagen wound biofilm model (≥30 h), the concentration of ammonia also stabilized. In contrast, the concentration of hydrogen cyanide continued to gradually increase throughout the remaining analysis time. Again, this is in contrast to that reportedly seen in planktonic culture [31], whereby production of hydrogen cyanide was maintained for only 1 to 4 h after the initial peak. These differences may result from changes in the metabolic activity associated with the biofilm mode of growth, coupled with the continuous supply of substrates for metabolism and removal of waste products within our model, which is more representative of the in vivo environment. The concentration of both hydrogen cyanide and ammonia remained at levels detectable by SIFT-MS throughout the 72 h sampling time, confirming the potential application of detection of these compounds as markers of *P. aeruginosa* presence that could usefully be exploited through development of rapid point of care diagnostic devices.

## Conclusions

The collagen wound biofilm model has been successfully developed and evaluated for the growth of steady-state biofilms under wound like conditions. We have demonstrated the potential of the collagen wound biofilm model for use in metabolomics studies, by characterising volatile metabolite production from *P. aeruginosa*, a clinically relevant pathogen associated with wound infection. Furthermore, the collagen wound biofilm model not only demonstrates the failure of biofilm eradication using a clinically relevant ceftazidime concentration, but also allows the evaluation of antimicrobial kinetics, clearly demonstrating the development of tolerance in the biofilm cultures during treatment of biofilms at both early and late stage development.

## Methods

### Preparation and maintenance of bacterial cultures

*P. aeruginosa* (NCIMB 10548) was maintained on beads (Pro-Lab Diagnostics, Birkenhead, UK) at − 80 °C, resuscitated as required on Tryptone Soya Agar (TSA) (Oxoid Ltd. Basingstoke, UK) and incubated aerobically at 37 °C. Working cultures were stored on sealed plates at 4 °C.

### Collagen coating of glass slide coupons

The collagen gel matrix was prepared based on the method described by Werthén et al. (2010) by preparing a collagen solution (2.0 mg mL^− 1^) in simulated wound fluid (SWF) [18, 21, 25, 26]. The SWF comprised of equal volumes of fetal bovine serum (Life Technologies Limited, Paisley, UK) and a solution of 0.1% bacteriological peptone (Oxoid Ltd. Basingstoke, UK) and 0.85% sodium chloride (Fisher Scientific Limited, Loughborough, UK). High concentration collagen (type I) from rat tail in 0.02 M Acetic Acid (Corning Incorporated, Wiesbaden, Germany) was neutralized to pH 7 with 1 M sodium hydroxide according to manufacturer instructions and diluted to the desired concentration with SWF. For example, to prepare 10 mL of 2.0 mg mL^− 1^ collagen solution in SWF from a collagen stock solution of 9.59 mg mL^− 1^; 48 μL of ice cold sodium hydroxide was added to 7.866 mL of ice cold SWF and mixed. On ice, 2.086 mL of ice cold collagen stock solution was then added and the solution mixed gently. Sterile glass microscope slides measuring 76 mm × 26 mm were coated with 1.5 mL of neutralized collagen solution (2.0 mg mL^− 1^), resulting in a depth of 760 μm. Collagen coated slides were incubated at 37 °C for 1 h to allow polymerization of the three-dimensional collagen matrix.

### Preparation of bacterial cultures

Overnight plate cultures (18–24 h) were used to prepare a suspension of *P. aeruginosa* in 10 mL SWF, adjusted to an OD_620nm_ of 0.20, equivalent to 2 × 10^8^ cfu mL^− 1^. One milliliter of the test suspension was used to inoculate each collagen coated microscope slide (individually housed in sterile petri dishes) and incubated at 33 °C for 2 h to allow initial adherence of bacterial cells.

### Growth of bacterial biofilms within the collagen wound biofilm model

**Fig. 7:**
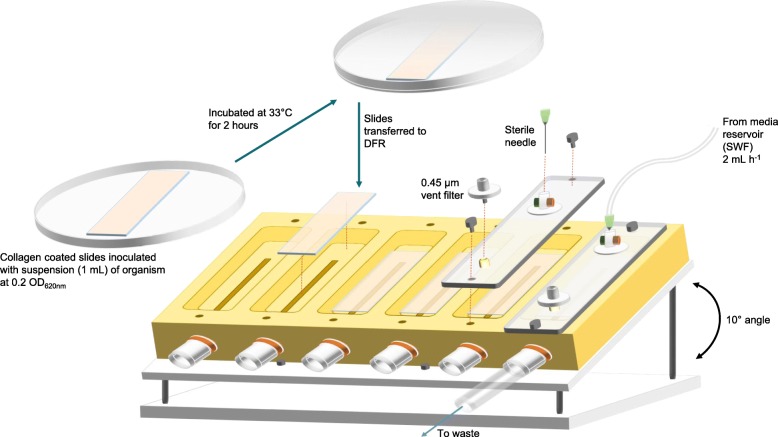
In vitro collagen wound biofilm model, comprising a drip flow reactor set-up incorporating collagen coated slide coupons, showing assembly with tubing connected to simulated wound fluid reservoir and waste collection

Following incubation, the inoculated slides were rinsed gently three times with 1 mL of sterile SWF to remove planktonic cells, and carefully transferred to individual channels within the drip flow reactor (Biosurface Technologies Corporation, Bozeman, MT, USA). Sterile silicone tubing (3 mm ID) was used to connect a 500 mL Duran bottle containing the sterile SWF medium to the miniert valve lid inlets via a 23 Gauge 1.25 in sterile needle. Waste was collected in 250 mL Duran bottles connected to the waste outlet ports using lengths of sterile silicone tubing (8 mm ID). The reactor was incubated at 33 °C to simulate average wound bed temperature [61] for 48–72 h at an angle of 10° to allow SWF to flow through the individual chambers of the model system (Fig. 7). The collagen wound biofilm model was continuously perfused with SWF at a flow rate of 2 mL hr.^− 1^ for the duration of incubation to simulate the flow of a moderately exuding wound (50 mL over 24 h) [62].

### Imaging of bacterial biofilms and collagen matrix

During preparation of collagen gel matrices for imaging experiments, a #1 glass cover slip measuring 18 mm × 18 mm was included between the microscope slide and collagen gel layer. The inclusion of a coverslip facilitated the sampling of a suitable sized section of the biofilm for microscopy. This was removed by cutting the collagen around the cover slip with a bespoke square stainless steel cutter and lifting away the coverslip supporting the biofilm sample with the aid of a scalpel blade.

### Scanning Electron microscopy (SEM)

Biofilm samples and an un-inoculated collagen gel control were fixed in 4% Glutaraldehyde (Sigma-Aldrich Company Limited, Gillingham, UK) in a 0.1 M phosphate buffer for 1 h at room temperature. This was rinsed in phosphate buffered saline (PBS) and dehydrated with increasing concentrations of ethanol and hexamethyldisilazane (Acros Organics, Geel, Belgium), air dried in a fume hood, mounted and gold sputter coated. A FEI Quanta 650 FEG scanning electron microscope operating at 10 kV was used to examine the samples.

#### Confocal scanning laser microscopy

Biofilm samples were stained using the FilmTracer LIVE/DEAD Biofilm Viability Kit (Fisher Scientific UK Limited, Loughborough, UK) according to manufacturer’s instructions: 3 μL each of Component A (SYTO 9 green fluorescent nucleic acid stain 3.34 mM in DMSO) and Component B (Propidium iodide 20 mM in DMSO) were added to 1 mL of sterile filtered deionized water to prepare a working solution. Three hundred microliters of the prepared staining solution was gently added to the biofilm surface. Samples were incubated at room temperature for 30 min in the dark. Following incubation samples were rinsed gently with sterile filtered deionized water to remove excess stain and imaged using the × 40 oil objective of the Leica DMi8 Inverted microscope with confocal scanner (Leica Microsystems (UK) Ltd., Milton Keynes, UK). Confocal Z-stack scans were exported to Fiji [63] for image processing.

### Enumeration of bacterial biofilms

Bacterial biofilms were sampled at 0, 3 and 6 h, then every 6 h until 48 h and finally at 72 h. At each time point a coated slide was aseptically removed from the reactor and the entire collagen layer containing the bacterial biofilm scraped into a 50 mL falcon tube using a sterile L-shaped scraper, while rinsing with 3 × 1 mL PBS. Two milliliters of 500 μg mL^− 1^ collagenase solution (Life Technologies Limited, Paisley, UK) was added to the tube, mixed and incubated at 37 °C for 20 min and then mixed by vortexing and incubated for a further 20 min. The resulting suspension was disrupted by sonication in a water bath (Fisherbrand FB11078, Fisher Scientific Limited, Loughborough, UK) at 35 kHz for 5 min. Collagenase solution was washed from the bacterial cells; whereby the suspension was centrifuged at 4000 x g, the supernatant discarded and pellet re-suspended in 10 mL PBS a total of two times. Bacterial density (cfu slide^− 1^) was determined by serially diluting in PBS and spiral plating (Whitley Automated Spiral Plater, Don Whitley Scientific Limited, Bingley, UK) on to TSA. Colonies were counted after 24 h incubation at 37 °C.

### Antimicrobial susceptibility – broth microdilution

The antimicrobial susceptibility of *P. aeruginosa* NCIMB 10548 to ceftazidime was tested based on the broth microdilution method described by BS EN ISO 20776-1:2006 [45]. Ceftazidime was prepared at concentrations ranging from 256 mg L^− 1^ to 0.25 mg L^− 1^. Fifty microliters of each antibiotic concentration and a control of 0 mg L^− 1^ of ceftazidime were dispensed in triplicate into wells of a 96 well multi-well plate. Overnight plate cultures were used to prepare a standardized suspension (1 × 10^6^ cfu mL^− 1^) of *P. aeruginosa* NCIMB 10548 in 10 mL Muller-Hinton Broth (MHB) (Oxoid Ltd. Basingstoke, UK). Fifty microliters of the inoculum suspension were added to each of the wells of the multi-well plate containing 50 μL of MHB or MHB with ceftazidime. The resulting final inoculum was approximately 5 × 10^5^ cfu mL^− 1^ and final ceftazidime concentrations ranged from 128 mg L^− 1^ to 0.125 mg L^− 1^, plus antibiotic free controls. Additionally, three wells were prepared containing 100 μL of MHB only as un-inoculated negative controls. The multi-well plate was then incubated at 37 °C for 18 h. The inoculum suspension was serially diluted and 100 μL spread plated on to TSA and incubated at 37 °C overnight to confirm appropriate inoculum preparation. Following incubation each well of the plate was visually inspected to identify turbidity to indicate growth of the test organism by comparison to the controls. Agar plates were counted after 18–24 h to confirm inoculum density was within the required range.

### Antimicrobial susceptibility – biofilms

Biofilm cultures were grown on collagen coated slides in the drip flow reactor system as described above. Ceftazidime treatment was started after either 6 h, when cultures are at an early stage of biofilm development, or 30 h of continuous culture, when biofilms are established and maturing. Residual SWF was drained via the tubing and the media reservoir refilled with fresh SWF containing ceftazidime at 40 mg L^− 1^. Biofilms were sampled periodically as described above to determine the effect of ceftazidime treatment on biofilm density over time.

### SIFT-MS analysis of bacterial biofilms

Volatile compounds were sampled from the headspace of collagen wound biofilm cultures by connecting the heated sample inlet of the selected ion flow tube mass spectrometry (SIFT-MS) instrument (Voice200Ultra, Syft Technologies, Christchurch, NZ) to the reactor channel via a length of PEEK tubing. To connect the PEEK tubing, bespoke modified biofilm reactor lids with an additional valve port were used. The SIFT-MS instrument was operated in selected ion mode (SIM) using the H_3_O^+^, NO^+^ and O_2_^+^ reagent ions to quantify hydrogen cyanide and ammonia concentrations in the biofilm headspace throughout the 72 h growth and development period.

### Data analysis

Biofilm enumeration data were analyzed by performing t-tests comparing specific time points of interest using Graphpad Prism 7 (GraphPad Software Inc., California, USA). SIFT-MS data for each independent *P. aeruginosa* biofilm were extracted using MATLAB R2018a (The MathWorks, Inc., Natick, Massachusetts, United States). In order to remove signal noise from the data the mean concentration of each compound was calculated for each hour of analysis.

## Data Availability

The datasets used and/or analysed during the current study are available from the corresponding author on reasonable request.

## Abbreviations

CFU: Colony Forming Units
EPS: Extracellular Polymeric Substances
MHB: Mueller-Hinton Broth
MIC: Minimum Inhibitory Concentration
NCIMB: National Collection of Industrial Food and Marine Bacteria
OD: Optical Density
PBP3: Penicillin Binding Protein 3
PBS: Phosphate Buffered Saline
ppb: Parts Per Billion
SEM: Scanning Electron Microscopy
SIFT-MS: Selected Ion Flow Tube Mass Spectrometry
SIM: Selected Ion Mode
SWF: Simulated Wound Fluid
TSA: Tryptone Soya Agar
